# Supplementary material for: Exploring Support Provided by Community Managed Organisations to Address Health Risk Behaviours Associated with Chronic Disease among People with Mental Health Conditions: A Qualitative Study with Organisational Leaders
Source: Int J Environ Res Public Health. 2022 May 2;19(9):5533. doi: 10.3390/ijerph19095533 (PMC9105164; doi:10.3390/ijerph19095533)
Supplement: Supplementary file 1 [file ijerph-19-05533-s001.zip › ijerph-1647968-supplementary.pdf]

Supplementary Table S1: Interview guide for the semi-structured interviews.

Item label	Item	Response options
**Organisational Capacity questions**		
MH CMO Status	To start off with I want to ask you a question to determine what category of CMO *name of organisation* would fall into.<br><br>Which of the following does your organisation provide?	1. Mental health services or programs only<br>2. Some mental health services or programs, as well as other programs for different consumer groups<br>3. No specific mental health services or programs, but the organisation has made adjustments for mental health consumers<br>4. None of the above
Intro to OC questions	Just a reminder that when we are talking about the support provided to consumers; we are talking only about support provided to adult consumers with a mental health condition.<br><br>You may remember completing our online survey last year about the support your CMO provides to address the health behaviours of consumers with a mental health condition accessing your CMO. One of the key findings from the survey was that the level of support provided to consumers differed by health behaviours. As such, we would like to further explore what type of support is being provided for each behaviour separately. These health behaviours include: tobacco smoking, nutrition, alcohol consumption, physical activity and sleep.<br><br>I will also ask you about what allows your organisation to provide this support. I will ask you to think about things at an organisational level and a staff level. An organisation level include things that operate from the top-down. These could be things at a senior management level that impact the support provided to address health behaviours. This could include things like developing action plans and goals for the organisation regarding health behaviour support or planning and conducting evaluations of the support provided for example.<br><br>Staff level refers to things that operate on a day to day level. Including managerial and other staff, including peer workers. This can include things like staff having access to information and resources around providing preventive	N/A

	support and having adequate levels of staffing for example.	
**Smoking**		
Smoking: current support	First, I would like to ask you about tobacco smoking.<br><br>Can you tell me about the support your organisation provides to help consumers reduce or quit tobacco smoking?<br><br>Q1a. How would you rate your organisation's involvement in supporting consumers to reduce or quit smoking, on a scale of 1 to 10, with 10 being the highest level of involvement? [*What allows your organisation to provide support at this level?]*<br><br>Q1b. At an organisational level, what allows your CMO to provide this support?<br><br>Q1c. At a staff level, what allows your CMO to provide this support?	Open response
**Nutrition**		
Nutrition: current support	Now I will ask you about nutrition.<br><br>Can you tell me about the support your organisation provides to help consumers improve their nutrition?<br><br>Q2a. How would you rate your organisation's involvement in supporting consumers to improve their nutrition, on a scale of 1 to 10, with 10 being the highest level of involvement? [*What allows your organisation to provide support at this level?]*<br><br>Q2b. At an organisational level, what allows your CMO to provide this support?<br><br>Q2c. At a staff level, what allows your CMO to provide this support?	Open response
**Alcohol consumption**		
Alcohol: current support	Q3. Can you tell me about the support your organisation provides to help consumers reduce their alcohol consumption?<br><br>Q3a. How would you rate your organisation's involvement in supporting consumers to improve their nutrition, on a scale of 1 to 10, with 10 being the highest level of involvement? [*What allows your organisation to provide support at this level?]*	Open response

	Q3b. At an organisational level, what allows your CMO to provide this support?<br><br>Q3c. At a staff level, what allows your CMO to provide this support?	
**Physical inactivity**		
Physical inactivity: current support	Q4. Can you tell me about the support your organisation provides to consumers to improve physical activity?<br><br>Q4a. How would you rate your organisation's involvement in supporting consumers to improve their nutrition, on a scale of 1 to 10, with 10 being the highest level of involvement? [*What allows your organisation to provide support at this level?]*<br><br>Q4b. At an organisational level, what allows your CMO to provide this support?<br><br>Q4c. At a staff level, what allows your CMO to provide this support?	Open response
**Sleep**		
Sleep: current support	Can you tell me about the support your organisation provides to consumers to improve sleep?<br><br>Q5a. How would you rate your organisation's involvement in supporting consumers to improve their nutrition, on a scale of 1 to 10, with 10 being the highest level of involvement? [*What allows your organisation to provide support at this level?]*<br><br>Q5b. At an organisational level, what allows your CMO to provide this support?<br><br>Q5c. At a staff level, what allows your CMO to provide this support?	Open response
End of interview		
